# Bathing adaptations in the homes of older adults (BATH-OUT): protocol for a feasibility randomised controlled trial (RCT)

**DOI:** 10.1136/bmjopen-2016-013448

**Published:** 2016-10-06

**Authors:** Phillip J Whitehead, Marilyn James, Stuart Belshaw, Tony Dawson, Miriam R Day, Marion F Walker

**Affiliations:** 1 Division of Rehabilitation and Ageing, University of Nottingham, Nottingham, UK; 2 Nottingham City Council, Nottingham, UK

**Keywords:** Older Adults, Bathing, Quality of Life, Housing Adaptations & Modifications

## Abstract

**Introduction:**

The Care Act 2014 has placed a responsibility on local authorities in England to provide services that prevent deterioration and minimise the use of other health and social care services. Housing adaptations have been identified as 1 of the 10 most promising prevention services for older adults, with bathing adaptations being the most requested. However, many local authorities have lengthy waiting times which may increase costs, reduce effectiveness and reduce the preventive effect. There is no robust evidence of the effect of these adaptations on: health, well-being and functional ability.

**Methods and analysis:**

This is a feasibility randomised controlled trial (RCT) with nested qualitative interview study. The RCT will recruit between 40 and 60 people who have been referred for an accessible showering facility, and their carers, from 1 local authority in England. They will be randomised to either usual adaptations (∼3-month wait) or immediate adaptations (no wait). The primary outcome is the feasibility of conducting a powered study. The outcomes assessed will be: health and social care-related quality of life, independence in activities of daily living and bathing, falls and use of health and social care services. Outcomes will be assessed at 3 and 6 months. Preliminary health economic feasibility will be established.

**Ethics and dissemination:**

Favourable ethical opinion was provided by the Social Care Research Ethics Committee (reference number 16/IEC08/0017). The results of this study will lay the foundations for a further powered study. This would investigate the effect of bathing adaptations on quality of life and whether increased waiting times are associated with poorer outcomes and increased costs. The results have further potential to inform trials of other housing or social care interventions using the novel waiting list control method. Dissemination will include peer-reviewed publications and presentations at national and international conferences.

**Trial registration number:**

ISRCTN14876332; Pre-results.

**Strengths and limitations of this study**

- This study will determine the feasibility of conducting a randomised controlled trial and health economic evaluation of bathing adaptations. Bathing adaptations are important preventive social care interventions.
- This use of a waiting list control has the potential to inform trials of other interventions in housing and social care settings.
- To the best of our knowledge, this is the first randomised study of major housing adaptations in the UK.
- This feasibility study will be conducted in a single site involving a shorter waiting list control period than we anticipate would be used in the main study.

## Introduction

### Background and rationale

An adaptation is defined as ‘any permanent alteration to a building carried out with the intention of making the building more suitable for a disabled person’;^1^ internationally, these may be referred to as ‘home modifications’.^2^ Bathing adaptations usually involve the removal of a bath and replacement with an accessible showering facility and are the most commonly requested adaptation.^3^ The Care Act 2014^4^ has placed a responsibility on local authorities to provide services which prevent or delay the need for care and support. In a review of national and international evidence on prevention in older people's services, Allen and Glasby^5^ reported that housing adaptations were 1 of the 10 ‘most promising’ interventions. They reported that housing adaptations can lead to: improved quality of life, reduced use of care services (such as homecare), postponed entry into residential care and reductions in falls. They are thus potentially associated with significant cost savings and better, preventive, outcomes for users and carers. However, despite the inclusion of housing adaptations in this review, the findings from studies that have investigated their effects are equivocal.

Major adaptations to bathing facilities are indicated when a person is unable to access the bath safely and/or independently and are recommended by occupational therapists when other bathing equipment is unsuitable. Removal of the bath and replacement with an accessible shower usually costs between £3000 and £4000; in England and Wales, a means tested government grant, the Disabled Facilities Grant (DFG), is available to assist with the cost of these adaptations.^6^ However, there are often lengthy delays^7^ and in local authority areas, waiting times can be in excess of 1-year and sometimes up to 2 years.^8^ Delays in provision of adaptations are reported to increase the risk of falls, hospitalisation^9^ and lead to increased costs due to further care being required during the wait.^10^ Care and Repair England^11^ have estimated that a delay of 1-year in providing a housing adaptation to an older person can increase homecare costs by £4000; this is comparable with the cost of providing a bathing adaptation. A recent survey revealed that 96% of occupational therapists believed that adaptations led to reductions in the need for social care services.^8^ It is seemingly counterintuitive to delay such interventions.

Older adults are the principal users of social care services.^12^ The onset of bathing disability has been shown to be a significant event in the disabling process for older adults. A cohort study in the USA followed 754 non-disabled adults, aged over 70, every month for 6 years with regard to their difficulties in completing particular activities of daily living (ADL).^13^ Those who developed a disability in bathing were five times more likely to develop a disability in another activity of daily living the following month. This demonstrates that the onset of disability in bathing may be a seminal point in the life of an older adult, acting as a warning point for the onset of further disability. Gill *et al*^13^ concluded that programmes need to restore and maintain independent bathing for older adults, in order to prevent further deterioration in their ability to function. Such programmes may thus have a strong preventive effect.

There is a lack of quantitative evidence of housing adaptations and bathing adaptations in particular. We are aware of only one randomised controlled trial (RCT) of housing adaptations which was conducted in New Zealand.^2^ This study randomised over 800 households to receive minor adaptations, including rails and step alterations, particularly directed towards reducing injurious falls. The adaptations package led to a 26% reduction in injuries caused by falls at home that required medical intervention. However, this study focused only on minor adaptations rather than more extensive home adaptations such as bathing facilities. Two further studies on adaptations have measured ability to perform ADL and perceived health status, respectively.^14^ ^15^ One study used a longitudinal before-and-after design and included different types of adaptations, with the authors reporting a decrease in dependence in bathing following the adaptations.^14^ The other study provided housing improvements, including bathing adaptations, with improvements in mental well-being reported following the intervention.^15^ However, the absence of a control group in both studies means that the underlying effect is unknown.

Findings from qualitative research suggest that adaptations are appreciated by service users and carers who believe that they have led to improvements in their health and well-being. For example, semistructured interviews were completed with 104 recipients of major adaptations drawn from 7 areas in England and Wales. The findings were that there were improvements in the physical and mental health of the users and their family members. Furthermore, findings from postal surveys have revealed extremely high levels of satisfaction with housing adaptations and self-reports that the adaptations led to improvements in quality of life.^9^ ^16^

The evidence for cost savings associated with adaptations is mixed. A housing adaptations review concluded that adaptations could lead to large-scale cost savings in residential care and the healthcare costs associated with accidents such as falls.^9^ However, it also reported that the evidence for cost savings associated with homecare for older adults was less clear. The evidence cited in the review was primarily drawn from case studies and examples from local authorities.

Although studies have suggested promising outcomes, there are limitations with the findings from previous research. First, studies have focused on disparate populations, including older and younger adults and different types of adaptations. This heterogeneity of study population and intervention type is likely to be diluting the effect. Second, studies have focused on different outcomes using a myriad of outcome measures, making synthesis of the findings problematic. Third, studies focusing on major or bathing adaptations are methodologically weak, with small samples, without control groups. There is therefore a paucity of high-quality quantified evidence of the effect of housing adaptations on quality of life and functional ability.

### Why is this study needed?

Although bathing adaptations may be perceived to be costly, there is no robust research evidence of their cost-effectiveness. When the costs of an intervention are evaluated in relation to improvements in quality and length of life, as advocated in the NICE reference case,^17^ cost-effectiveness can be demonstrated. Randomised study designs are rare in social care settings, but they are believed to be the most robust method by which to compare the effects of one treatment over another,^18^ calculate the effect of interventions on quality of life (including quality-adjusted life years gained) and conduct robust cost-effectiveness analyses.^19^

### Research aim and objectives

The aim is to determine the feasibility of conducting a powered RCT (with waiting list control group) of bathing adaptations for older adults and their carers. A powered trial would investigate the effect of bathing adaptations on quality of life, perceived health status, functional deterioration and to examine whether routine waiting times are associated with poorer outcomes and increased costs. Specific objectives are to: recruit 40–60 participants to the study, recruit a minimum of 50% of those eligible, provide 70% of adaptations within the specified timescales, follow-up a minimum of 70% of participants at the 6-month time point and achieve a minimum of 80% completeness of data.

## Methods and analysis

### Study design and setting

This is a single-centre feasibility RCT with nested qualitative interview study. The RCT is a parallel group, two-arm trial with a 1:1 allocation ratio intervention: waiting-list control. Outcomes will be assessed by a researcher blinded to group allocation. The study will be conducted within one city council in England. The service has a dedicated Adaptations and Renewals Agency which coordinates and manages major adaptations (costing over £1000) for public sector (council owned) and private properties where a DFG is being used to fund or part-fund the adaptations.

### Participants

Participants will be adults, aged 65 or over, referred to the Adaptations and Renewals Agency, by a social care occupational therapy team member, for provision of an accessible showering facility. Exclusion criteria are: being referred for an accessible showering facility plus one or more other adaptations (eg, hoist, ramp, lift), priority ‘A’ referrals (those which are being ‘fast-tracked’ based on clinical assessment). We will also exclude adaptations involving provision of or alterations to baths which are rarely provided within the authority.

Where a participant has a carer, they will also be approached for informed consent to take part in the study. We will take a broad definition of ‘carer’ which will be led by the service user and carer's views of their role. This will encompass people who provide practical and/or emotional support, those who assist with personal care and those who do not. NB where a service user consents to take part in the study, but a carer declines then the service user will still be eligible to participate.

### Intervention and comparator

The intervention is the provision of an accessible showering facility. This usually involves the removal of an existing bath and replacement with a flush floor antislip walk in ‘level access’ shower (which may also be termed a ‘wet room’). It may also include an easy access shower or the alteration of an existing shower cubicle to make it more accessible. Participants in both groups will receive this intervention; however, they will be randomised to either:

- Usual adaptations service (waiting-list control group): Those randomised to the control group will receive the usual routine service provided by the Adaptations and Renewals Agency. This involves being allocated to a project officer to begin planning the accessible showering facility after a 3-month wait.
- Intervention (no waiting list): Those randomised to the intervention group will be allocated to a project officer begin planning the accessible showering facility immediately and will not go onto the routine waiting list.

It is possible that participants may choose to discontinue with their adaptations after randomisation (ie, not to have the accessible showering facility installed). We anticipate that these instances will be rare. We will record these instances as part of our assessment of feasibility.

### Outcomes

The main outcome for the study is to determine the feasibility of conducting a larger, powered study. This will be a composite of: whether the eligibility criteria are realistic; whether users and carers are willing to be randomised; the study attrition rate; whether the adaptations can be completed within 4–6 weeks of allocation to a project officer (in both groups); the suitability and sensitivity of outcome measures; the most suitable outcome measure for use in the main study; the feasibility of collecting the data on costs and health and social care use.

The service user outcomes to be assessed, at 3 and 6 months postrandomisation, will be: health and social care-related quality of life, perceived physical and mental well-being, personal ADL, independence in bathing, perceived difficulty in bathing, perceived risk of falling, falls, number of care support hours, health and social care service usage. The outcome measures which will be used are: EuroQol EQ5D-5L,^20^ Adult Social Care Outcomes Toolkit (ASCOT),^21^ Short-Form 36 (physical and mental component summaries),^22^ Barthel index^23^ (bathing question analysed as a separate outcome), 0–100 scale for perceived difficulty in bathing and the Falls-Efficacy Scale.^24^ A purposely designed questionnaire will gather information on the use of other health and social care services, with particular emphasis on the use of homecare and residential care.

**Figure 1:**
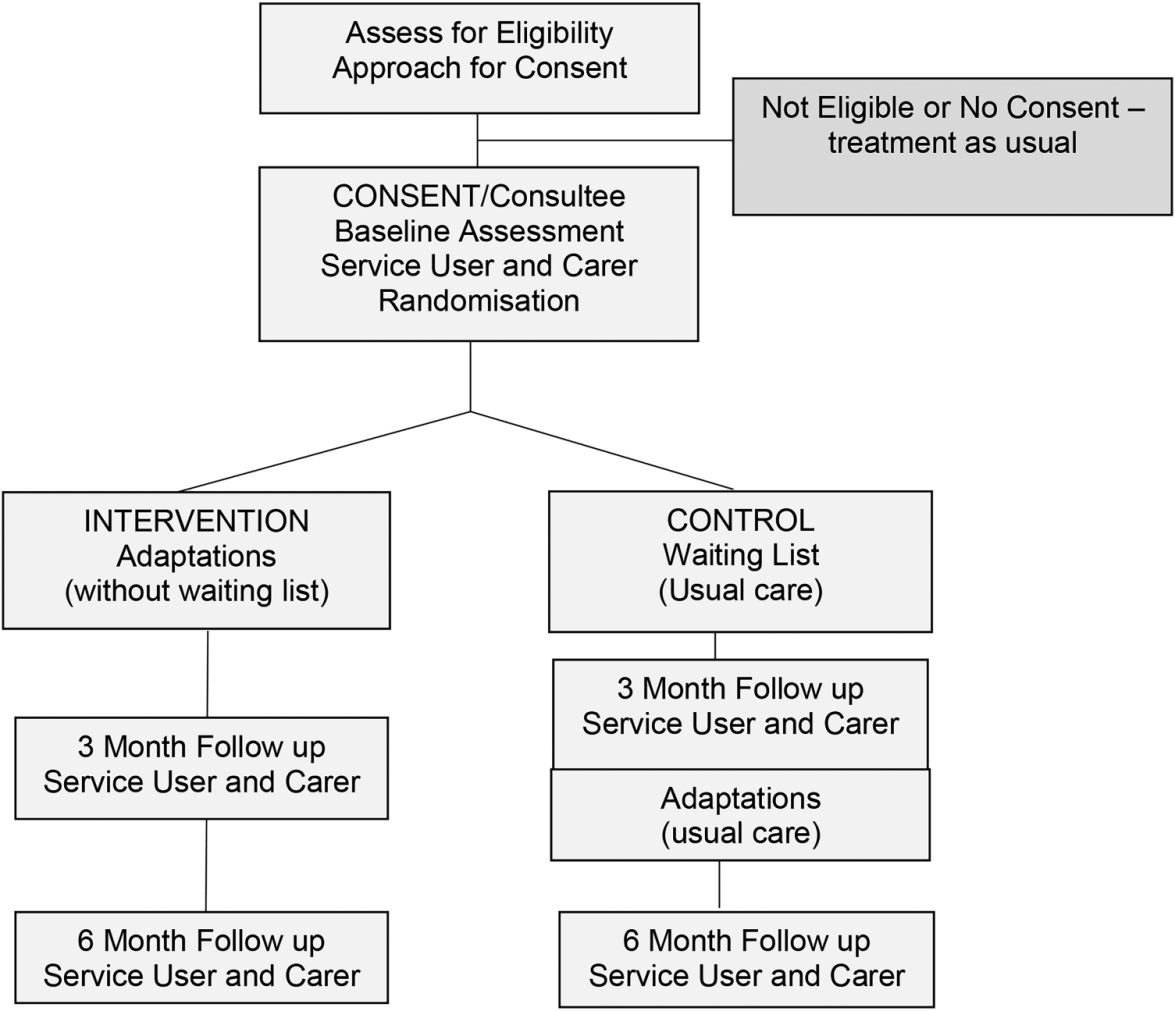
Flow of participants through the study.

The carer outcomes to be assessed, at 3 and 6 months postrandomisation, will be: health-related quality of life, perceived physical and mental well-being and caregiver strain. The outcomes measures which will be used are: EuroQol EQ5D-5L,^20^ Short-Form 36 (physical and mental component summaries)^22^ and Caregiver Strain Index.^25^ We will also gather data on the carers' use of health and social care services. The timeline and proposed flow of participants through the study is shown in figure 1.

### Qualitative interviews

Semistructured qualitative interviews will be completed with up to 20 service user participants and 10 carer participants. The aim of the interviews is to explore and identify factors associated with the bathing adaptations which may inform the design of a further trial. Specific objectives are: (1) to identify the factors that precipitated the need for bathing adaptations for older adults and their carers; (2) to identify specific facilitators and barriers associated with the provision and timing of the bathing adaptations; (3) to identify any aspects of study participation that could be improved or enhanced to inform the design of a further study.

Interviewees will be purposively sampled for a variety of characteristics from the intervention and waiting list control groups in order to gain a range from both groups in the feasibility RCT. Service users will be sampled to include: men and women, those who live alone and those who live with support, those who are in receipt of ongoing social care services and those who are independent. Carers will be sampled to include: men and women, those of the same generation to the person they provide care for and a different generation, those who provide assistance with personal care activities and those who do not. Interviews will be analysed using thematic analysis.^26^

### Concomitant treatments

There are no known issues with concomitant treatments and no treatments will be excluded. It is expected that participants in both groups will receive a range of input from other health and social care services. Information will be kept on the participant's use of other acute and community services and will be reviewed as part of the health and social care resource use data.

### Intervention delivery and cost collection

Information will be gathered on the costs of the intervention and the timescales to deliver the adaptations in both groups. Deviations in the planned timescales for delivery will be recorded and reasons will be recorded qualitatively.

### Sample size and recruitment strategy

For a feasibility study, no formal sample size calculation is required. The aim is to recruit between 40 and 60 participants (20–30 in each arm of the trial) to test the randomisation process and the feasibility of delivering the intervention in the proposed timescales. This target should allow us to collect sufficient information on the suitability and sensitivity of the outcome measures for use with this population and the SDs of the measures to inform a sample size calculation for a further study. The median sample size for feasibility UK feasibility trials has been reported at 36^27^ which is broadly consistent with the planned minimum target.

The trial will recruit for 8 months. Current data from the trial site suggest that ∼15 service users per month will be eligible. All potentially eligible participants will be approached consecutively in the order in which they are referred to the Adaptations and Renewals Agency. If the maximum of 60 participants are recruited before the end of the 8-month period, recruitment will cease.

Participants will be enrolled into the study by a member of the research team. The process for obtaining participant informed consent will be in accordance with the REC guidance, and Good Clinical Practice (GCP) and any other regulatory requirements that might be introduced. Following a full explanation of the study by a member of the research team, the participant shall provide informed written consent before they can participate in the study. Where a consultee is required, they shall provide a recommendation as to whether they consider the person would have agreed to take part in the study, had they still had capacity to state their own preference. They will sign the consultee declaration, should they believe that person would have wished to take part in the study.

Randomisation will be generated online using a web-based randomisation programme http://www.sealedenvelope.com. Participants will be individually randomised in random varying block sizes (sized in order to deliver the adaptations appropriately). Randomisation will be stratified according to whether the property is publicly or privately owned. Randomisation will be at a ratio of 1:1 (immediate adaptations to waiting list control). Members of the research team will not have access to the allocation sequence.

Baseline assessments will be completed prior to randomisation. Follow-up assessment visits will be completed by a research assistant who is blinded to allocation. To minimise the risk of unblinding, prior to each contact, the participant will be reminded that the researcher who is to conduct their follow-up assessment is blinded. Additionally, the researcher will avoid entering the areas of the home where adaptations have been provided (ie, the bathroom). It is possible that participants may reveal their group allocation to the outcome assessors and any instances of this will be recorded by researchers as part of the assessment of feasibility; researchers will also be asked to make their ‘best guess’ as to the group allocation of the participants to determine whether blinding was successful. Other members of the research team and investigators will not be blinded to group allocation for the purpose of managing the trial and delivering the interventions. It will not be possible to mask participants.

### Data collection, management and analysis

Data will be collected in the participants' homes on a paper case report form (CRF) and will subsequently be entered onto a secure password protected, purposely designed electronic Microsoft Access database. Outcome data will be entered by the research assistant who collected the data (thus will be entered blinded to treatment allocation). Each participant will be assigned a trial identity code number, allocated at randomisation, for use on CRFs other trial documents and the electronic database.

CRFs will be treated as confidential documents and held securely in accordance with regulations. The investigator will make a separate confidential record of the participant's name, date of birth, local social care number and participant trial number to permit identification of all participants enrolled in the trial, in accordance with regulatory requirements and for follow-up as required.

When data collection is complete, a data quality check will be conducted in duplicate by two researchers and a 10% sample of the database will be checked against the original paper CRF. Steps will be taken to minimise missing data by personal contact throughout the study period from the investigator and every attempt will be made to locate participants for follow-up. Outcome data will be collected in person by a research assistant to minimise the amount of missing data. For each outcome measure used where data are missing, an imputed average will be used for items where <10% of the overall measure is missing. Where more than 10% of a measure is missing, the entire measure will be coded as missing, unless the scoring criterion for that measure stipulates an alternative approach. We will not collect any further data for participants who withdraw from the study, but we will retain all data collected up until the point of withdrawal.

The main end point for the study is to determine the feasibility of conducting a larger, powered study. Descriptive statistics will be used for this analysis, based on analysis of the trial screening and recruitment log, loss to follow-up, and analysis of the qualitative interview data. Analysis of outcome data will be by intention to treat, and participants will be analysed according to their treatment assignment irrespective of whether they completed the treatment. It will not be possible to collect any outcome data for those who discontinue participation in the study. The data collected from the outcome measures in the trial will be presented using summary statistics and any differences between the arms will be calculated at 3 and 6-month follow-ups, along with the 95% CIs. These data will be used to inform a sample size calculation, treatment effect estimate and to determine the appropriateness of these measures for use in a larger, powered study. Assistance from a statistician will be available as required.

The pilot economic analysis will be conducted from a health service and societal perspective. It will measure service user outcomes using the EQ5D-5L and use clinical outcomes where appropriate. Detailed resource costing will be undertaken from a health and social care service, user and societal perspective. As such, a cost profile will be calculated for each arm of the trial. This will enable the study results to be reported in terms of cost utility and cost-effectiveness. An incremental cost-effectiveness ratio (ICER) and cost-effectiveness acceptability curves (CEACs) will be produced for the intervention versus usual care, including the joint uncertainty in differential costs and effects from the cost-effectiveness plane. The ICER provides a ratio measure of increment costs and effects of the intervention over usual care. The CEAC use probabilistic analysis to provide a measurement of probability or thresholds showing the various levels of confidence from 0 to 100 (0–1 in terms of probability) of the intervention being cost-effective at a given cost. It should be noted that any reporting of such data in this study are only a guide to any future potential evaluation and it is the proof and testing of the methods that will be the main focus of the health economic analysis not the final results in themselves.

### Safety monitoring and adverse events

We are not anticipating any adverse events as part of this intervention which is an earlier provision of a routine intervention, thus we will not record any as part of this study. However, we will collect information from participants, including hospital admissions and falls during all follow-up visits. As this is a feasibility trial, we will not convene a data monitoring committee. A trial advisory group is in place and includes experienced researchers, social care staff, third sector groups and public and patient representatives.

## Dissemination

To the best of our knowledge, this is the first RCT of any type of housing adaptation in the UK. It is also the first RCT of bathing adaptations specifically. We believe that this is indicated due to the possible particular preventive effect which may apply to bathing adaptations specifically. Although housing adaptations have been identified as 1 of the 10 most promising prevention service for older adults,^5^ there is a paucity of high-quality evidence of prevention effect on the use of other services, particularly homecare and residential care and health and social care-related quality of life. This study will provide the foundations for a further, appropriately powered study to investigate this. The findings will be relevant to researchers, clinicians, commissioners, service users and carers.

We plan to disseminate our findings through presentations at national and international social care and occupational therapy conferences, and we will submit findings for publication in a peer-reviewed academic journal.
